# Construction and analysis of a high-density genetic linkage map in cabbage *(Brassica oleracea* L. var. *capitata*)

**DOI:** 10.1186/1471-2164-13-523

**Published:** 2012-10-03

**Authors:** Wanxing Wang, Shunmou Huang, Yumei Liu, Zhiyuan Fang, Limei Yang, Wei Hua, Suxia Yuan, Shengyi Liu, Jifeng Sun, Mu Zhuang, Yangyong Zhang, Aisong Zeng

**Affiliations:** 1 Institute of Vegetables and Flowers, Chinese Academy of Agricultural Sciences, Key Laboratory of Biology and Genetic Improvement of Horticultural Crops, Ministry of Agriculture, P.R.China. Beijing, 100081, China; 2 Institute of Oil Crops Research, Chinese Academy of Agriculture Sciences, Key Laboratory for Biological Sciences of Oil Crops, P.R.China. Ministry of Agriculture, Wuhan, 430062, China

**Keywords:** Cabbage, *Brassica*, Genetic linkage map, SSR, SNP, Genome

## Abstract

**Background:**

*Brassica oleracea* encompass a family of vegetables and cabbage that are among the most widely cultivated crops. In 2009, the *B. oleracea* Genome Sequencing Project was launched using next generation sequencing technology. None of the available maps were detailed enough to anchor the sequence scaffolds for the Genome Sequencing Project. This report describes the development of a large number of SSR and SNP markers from the whole genome shotgun sequence data of *B. oleracea*, and the construction of a high-density genetic linkage map using a double haploid mapping population.

**Results:**

The *B. oleracea* high-density genetic linkage map that was constructed includes 1,227 markers in nine linkage groups spanning a total of 1197.9 cM with an average of 0.98 cM between adjacent loci. There were 602 SSR markers and 625 SNP markers on the map. The chromosome with the highest number of markers (186) was C03, and the chromosome with smallest number of markers (99) was C09.

**Conclusions:**

This first high-density map allowed the assembled scaffolds to be anchored to pseudochromosomes. The map also provides useful information for positional cloning, molecular breeding, and integration of information of genes and traits in *B. oleracea*. All the markers on the map will be transferable and could be used for the construction of other genetic maps.

## Background

*Brassicaceae* is a large family, consisting of approximately 340 genera and more than 3,350 species [1]. In addition to providing vegetable oil, vegetables, fodder and condiment, *Brassicas* are important sources for dietary fiber, vitamin C and other nutritionally beneficial factors such as anticancer compounds [2]. Cytogenetic research of the six cultivated species has shown that the group includes three diploid species, *B. rapa* (AA, 2n = 20), *B. nigra* (BB, 2n = 16), *B. oleracea* (CC, 2n = 18)], and three amphiploid species, *B. juncea* (AABB, 2n = 36), *B. napus* (AACC, 2n =38) and *B. carinata* (BBCC, 2n = 34)]. In addition, interspecific hybridization studies demonstrated that three diploid species contain the basic chromosome sets, while the amphiploid species contain hybridized and naturally doubled combinations of the three diploid species in a relationship that is referred to as U’s triangle [3]. The genome sizes of the diploid *Brassicas* and the allopolyploids are 529–696 Mb and 1068–1284 Mb respectively [4].

Long-term cultivation and artificial selection of *Brassica* crops have resulted in rich intraspecific morphological variations all of which are adapted for various cultivation conditions [5]. For instance, well-established vegetables of the *B. oleracea* species comprise a number of morphologically diverse crops, including cabbage (*B. oleracea* var. *capitata*), Brussels sprouts (*B. oleracea* var. *gemmifera*), kale (*B. oleracea* var. *acephala*), kohlrabi (*B. oleracea* var. *gongylode*), Chinese kale (*B. oleracea* var. *alboglabra*), broccoli (*B. oleracea* var. *italica*) and cauliflower (*B. oleracea* var. *botrytis*).

Cabbage (*B. oleracea* var. *capitata*) is considered to be a typical representative of the C genome of *Brassica* and the *B. oleracea* Genome Sequencing Project (BrGSP) was launched in 2009. The *B. oleracea* material that was used for the *de novo* sequencing was an advanced homozygous inbred line 02–12. The primary sequencing project has been completed and the findings will be published shortly. To anchor the assembled scaffolds to pseudochromosomes, a high-density genetic map based on sequence-tagged PCR-markers is required.

A high-density genetic map can also form the basis for quantitative trait loci mapping (QTL mapping), marker assistant selection (MAS), and functional gene positional cloning, and will be useful for functional genomics and genetic breeding studies. A comparison of the genetic maps of closely related species will contribute to an understanding of the origin of relationships among the *Brassica*s, and genetic maps can provide insights into genome organization and evolution through comparative mapping.

More than ten genetic linkage maps of *B. oleracea* have been constructed [6]. The early genetic maps used restriction fragment length polymorphism (RFLP) markers [7-9]. However, RFLPs requires a large amount of DNA and the procedure is inefficient and difficult to apply in breeding. With the invention of the polymerase chain reaction (PCR), a variety of PCR-based markers, such as simple sequence repeats (SSRs) were successively developed and became the preferred markers. SSRs require only small amounts of DNA, are easily generated by PCR, are amenable to high-throughput analysis, codominantly inherited, multi-allelic, highly polymorphic, abundant, and are evenly distributed in genomes [10]. SSRs have been extensively used in tagging qualitative genes and in dissecting the genetic bases of complex traits [11-13]. Recent developments in sequencing technology have simplified and accelerated the discovery of sequence variants, enabling the development of sequence-based markers including single nucleotide polymorphisms (SNPs) and insertion/deletion polymorphism (InDel) markers [14]. SNPs are the markers of choice for high-resolution genetic mapping and association studies because of their abundance and widespread distribution throughout the genome [15]. These third generation markers, however, have rarely been used for genetic linkage mapping in *B. oleracea*.

*B. oleracea* genetic maps are most often constructed using populations obtained from crosses between subspecies and varieties, and F_2_ populations that are not immortal [8,9]. F_2_ mapping populations are temporary and difficult to maintain for long term and comparative studies. To produce high-resolution genetic maps for future research, double haploid (DH) and recombinant inbred line (RIL) populations are more often used for mapping. However, to date, no studies have reported the use of a DH population for mapping between cabbage varieties*.*

We generated a double haploid (DH) population derived from an F_1_ cross between two advanced homozygous inbred lines, 01–88 and 02–12, by microspore culture. A number of SSR and SNP markers were developed using the whole genome shotgun sequence data from the BrGSP. These markers were then used to construct a saturated genetic map of the *B. oleracea* genome that could be used to anchor and orientate sequence scaffolds from the *B. oleracea* genome assembly.

## Methods

### Development of a mapping population and DNA isolation

Two diverse advanced homozygous inbred lines of cabbage, 01–88 and 02–12, were used as the parents to develop a doubled haploid (DH) mapping population containing 165 lines. The DH population was derived from the F_1_ by microspore culture [16] and contained lines with a wide variety of morphological traits.

Total DNA was isolated from the expanding leaves of three-week old plants using the modified cetyltrimethylammonium bromide (CTAB) method [17]. The genomic DNA samples were adjusted to 50 ng DNA/μl and preserved at −20°C until used as the templates for PCR amplification. Additional, leaf tissue was lyophilized for use in future experiments.

### Marker sources

The two marker types (SSRs and SNPs) were obtained from seven sources including genomic DNA sequence data and gene or EST databases (Table 1). A total of 3,378 SSR markers were developed from the cabbage sequence scaffolds and 2,200 SNP markers were developed by resequencing the other parent (line 01–88) of the *B. oleracea* mapping population. The 551 published SSR primer pairs derived from the genomic sequences of *B. rapa* (prefixed by Ra), *B. oleracea* (ol), *B. napus* (Na) and *B. nigra* (Ni), were obtained from the BrassicaDB database [18-21]. The FITO markers were designed by Iniguez-Luy [22]. A total of 62,567 ESTs in *B. oleracea* were downloaded from the National Center for Biotechnology Information (NCBI) for the identification and development of 1,080 EST-SSR markers [23]. Professor Liu Kede from Huazhong Agricultural University developed 268 markers with the data from the brassica.info database [6,24]. The data for the BAC end sequencing generated 292 SSR markers. The remaining 728 SSRs were derived from the *B. rapa* genome. A total of 8,497 primer sets were developed for the 6,297 SSR and 2,200 SNP markers and used to scan for polymorphisms between two parents. 

**Table 1:** Source of the sequences and primers that were used in this study

**Code of primers**	**Number of primers**	**Source of primers**
cl, sc	3378	Sequencing of *B. oleracea*
snap	2200	Resequencing of *B. oleracea*
Ra, ol, Na, FITO	551	Public *B. oleracea* markers [18-22]
BoE	1080	Associate researcher Zhuang in IVF CAAS [23]
BnGMS	268	Professor Liu in Huazhong Agricultural University [6,24]
brbac	292	BAC database of *B .rapa*
brsf	728	Sequencing of *B. rapa*
Total	8497	

### Analysis of molecular markers

#### SSR detection

**Figure 1:**
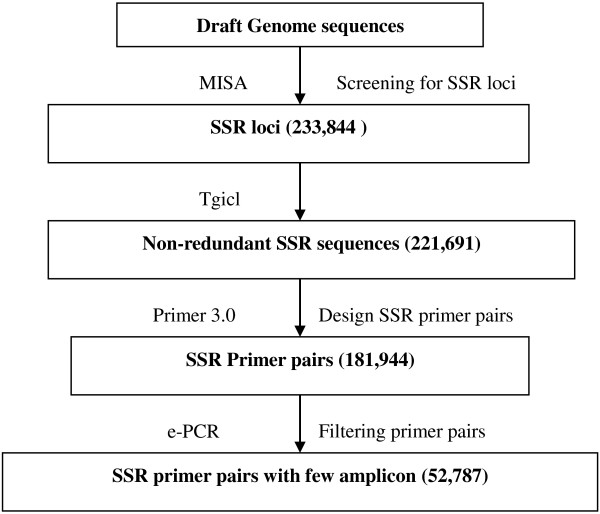
Flow chart of the process used to develop the cabbage SSR markers.

The process used for SSR markers development is presented in Figure 1. First, All SSRs were identified and selected using MISA [25] in the assembled sequences and expressed sequence tags. The minimum repeat units for MNRs (mononucleotides repeats), DNRs (dinucleotide repeats), TNRs (trinucleotide repeats), TTRs (tetranucleotide repeats), PNRs (pentanucleotide repeats), and HNRs (hexanucleotide repeats) were chosen to be 10, 6, 5, 5, 5, and 5, respectively. The maximum difference was 100 base pairs between two SSRs. 

#### SSR markers

First, the redundant SSR-containing sequences were identified by tgicl software ( http://compbio.dfci.harvard.edu/tgi/software/). Second, SSR primer pairs were designed using the Primer 3.0 program [26]. The primer lengths ranged from 18 to 23 nucleotides, with an optimum size of 20 nucleotides. The melting temperatures ranged from 50 to 65°C, with an optimum temperature of 55°C. The optimum GC content was set to 50%, with a minimum of 40% and a maximum of 60%. The predicted PCR products ranged from 200 to 300 bp. Third, the primer pairs were then filtered by e-PCR with default parameters [27]. All primers were synthesized by the Engineering Company, Shanghai, China.

DNA amplification of the SSR markers was carried out in volumes of 20 μl, containing 1 unit of Taq polymerase, 0.1 μM of each of the primers, 200 μM dNTPs, 2 μl 10 × buffer (Mg^2+^ 25 mM) and 50 ng of genomic DNA as templates. The PCR profile was as follows: initial 4 min at 94°C, then 35 cycles, each with 30 s DNA denaturation at 94°C, 30 s annealing at 55°C and 60 s extension at 72°C, and a final extension of 7 min at 72°C. The PCR was carried out in a GeneAmp PCR system 9700 (Applied Biosystems, California, US).

#### SNP markers

Firstly, a lot of SNPs (unpublished data) were identified between the 01–88 and 02–12 by soapsnp software ( http://soap.genomics.org.cn/soapsnp.html). Secondly, the SNP-contained sequences were extracted for primer design by SNAPER [28,29] using the default parameters. A total of 2,200 SNPs were transferred into SNAP(Single Nucleotide Amplified Polymorphisms) makers for genotyping via PAGE (PolyAcrylamide Gel Electrophoresis). PCR amplifications were performed in a volume of 20 μl containing 100 ng genomic DNA, 1 μl 10 × Taq buffer (Mg^2+^25 mM), 100 μM dNTPs, 0.05 μM each primer and 0.5 unit Taq DNA polymerase. The PCR profile was as follows: initial 4 min at 94°C, then 34 cycles, each with 15 s DNA denaturation at 94°C, 15 s at the appropriate annealing temperature (55-65°C) and 30 s extension at 72°C, and a final extension of 7 min at 72°C. The PCR was carried out in a GeneAmp PCR system 9700 (Applied Biosystems). The PCR products were separated on 8% non-denaturing polyacrylamide gels. After electrophoresis, the gels were stained as previously described [30].

### Linkage analysis and map construction

For map construction, the segregation in the DH population was analyzed for all the SSR markers that showed polymorphisms between the parental 01–88 and 02–12 lines. The markers contained two types of genotypic data: type A, the same as parent line 01–88, and type B, the same as parent line 02–12. Data that were unclear or missing for various reasons were indicated by ‘-’.

Markers which were reproducibly polymorphic between the parental lines were scored in the DH population. Linkage analysis and map construction were performed using JoinMap version 3.0 [26,31]. Linkage groups were identified in the LOD (logarithm (base 10) of odds) grouping threshold range of 5.0–10.0, and linkage groups were assigned as C01–C09, corresponding to the C-genome linkage groups of *B. napus*[32]. Maps were generated for each linkage group using a recombination frequency below 0.40 and LOD scores above 0.5 for all the markers within each linkage group. A ‘ripple’ procedure was performed after the addition of each marker and the ‘jump’ thresholds were set to 5. Recombination frequencies were converted to centiMorgans (cM) using Kosambi’s method for map-distance calculation [33].

### Marker distribution analysis

To evaluate whether the mapped markers were randomly distributed on the linkage map, the linkage groups were divided into 1, 2.5, 5, 10, 20, and 40 cM blocks, and the number of markers per block was counted. Observed frequencies of the number of markers per block were compared with the expected ones by performing a Chi-square test , using a Poisson distribution function, P(x) = e^-μ^μ^x^/x!, where x is the number of markers per block and μ is the average marker density in the consensus map. Average marker density (μ) was used to calculate the expected binomial frequencies for each marker class per block interval for all the linkage groups. The distribution of markers on the linkage groups was also evaluated separately for the SSR and SNP markers.

## Results

### Development of the mapping population

A total of 1,227 normal embryos were obtained from the 01-88 × 02-12 F_1_by microspore culture. Each bud generated approximately 70–120 embryos. After plant regeneration, 1,021 plants consisting of 170 haploids, 768 doubled diploids, 10 polyploids, and 73 chimeras were obtained. Finally, a DH population including of 165 individuals were obtained.

### Marker development

A total of 233,844 putative SSR sequences were identified from the cabbage assembled scaffold sequences. The frequencies of the different types of SSRs in the genome are listed in Table 2. Six different SSR repeat types were indentified, and of these the MNRs (163,621, 69.97%) were the most abundant and the HNRs were the least abundant (311, 0.13%). Among the DNRs, (AT)_n_ was the most abundant repeat motif (14.25%), followed by (AG)_n_ (7.95%) , (AC)_n_ (1.46%) and (CG)n(0.01%). All ten possible combinations of TNRs were observed in the SSRs. Among them, the (AAG)_n_ motif was the most common (1.83%), followed by the (AAT)_n_ (1.08%), (AGG)_n_ (0.61%), (AAC)_n_ (0.56%) and (AGT)_n_ (0.45%) motifs. Four main combinations of TTRs, four of PNRs and four of HNRs were also observed (Table 2). All the other repeat types were very rare in the *B. oleracea* genome.

**Table 2:** **Distribution of different types of SSRs in the *B. oleracea *genome**

**Motifs**	**Number**	**Percentage (%)**	**Total length (bp)**	**Average length (bp)**
Mononucletide	163621	69.97	1952399	11.93
A	158142	67.63	1872884	11.84
C	5479	2.34	79515	14.51
Dinucleotide	55336	23.66	968946	17.51
AT	33315	14.25	596070	17.89
AG	18593	7.95	322438	17.34
AC	3411	1.46	50220	14.72
CG	17	0.01	218	12.82
Trinucleotide	13080	5.59	254598	19.46
AAG	4281	1.83	80493	18.80
AAT	2535	1.08	63240	24.95
AGG	1434	0.61	26880	18.74
AAC	1313	0.56	22029	16.78
AGT	1062	0.45	19302	18.18
ACT	1004	0.43	18582	18.51
ACC	795	0.34	13245	16.66
AGC	271	0.12	4581	16.90
ACG	225	0.10	3708	16.48
CCG	160	0.07	2538	15.86
Tetranucleotide	1131	0.48	26960	23.84
AAAT	404	0.17	8916	22.07
AAAG	152	0.07	4060	26.71
AATT	110	0.05	2692	24.47
AAAC	94	0.04	2108	22.43
Others	371	0.16	9184	24.75
Pentanucleotide	365	0.16	14380	39.40
AAAAT	126	0.05	7710	61.19
AAAAC	31	0.01	830	26.77
AAAAG	25	0.01	690	27.60
AAACC	24	0.01	610	25.42
Others	159	0.07	4540	28.55
Hexanucleotide	311	0.13	11772	37.85
AGAGGG	23	0.01	1146	49.83
AAAAAC	16	0.01	510	31.88
AAGCCC	12	0.01	468	39.00
AAAAAT	12	0.01	636	53.00
Others	248	0.10	9012	36.34
Total	233844	100	3229055	13.81

A total of 1,026,766 SNPs were detected between 01–88 and 02–12, of these the A/G SNP type (597,814, 58.22%) was the most abundant and the G/C SNP type was the least abundant(72,115, 7.02%). While the A/C SNP type accounted for 21.96% (225,433) and the A/T SNP type accounted for 12.80%(131,404). A total of 2,200 SNP markers were transfered to SNAP markers for genotyping the mapping population.

A total of 1,096,647 EST were downloaded from NCBI. A total of 445,139 SSR loci were detected from the EST sequences (Table 3). The proportion for the MNRs and TNRs in *Brassica* EST sequences was higher than in *B.oleracea* genome sequences except AAT motif. The frequency of the most types of repeat units (DNRs, TTRs, PNRs and HNRs) in the genome was higher than that in ESTs except three motifs (AG, CG and AAAAAC). Especially AT motif frequency in the genome (14.25%) was much higher than that in ESTs (1.37%).

**Table 3:** **Distribution of the SSR frequency in *Brassica *EST sequences**

**Motifs**	**Number**	**Percentage (%)**	**Total length (bp)**	**Average length (bp)**
Mononucletide	339538	76.277	7614099	22.42
A	310831	69.828	7256120	23.34
C	28707	6.449	357979	12.47
Dinucleotide	55734	12.521	935952	16.79
AG	44403	9.975	756440	17.04
AT	6090	1.368	99192	16.29
AC	5186	1.165	79618	15.35
CG	55	0.012	702	12.76
Trinucleotide	48202	10.829	818475	16.98
AAG	17256	3.877	298896	17.32
AGG	7389	1.660	123783	16.75
ACT	5199	1.168	88563	17.03
ACC	3878	0.871	62421	16.10
AAC	3644	0.819	61962	17.00
AGT	3433	0.771	58869	17.15
AGC	2305	0.518	38754	16.81
ACG	2063	0.463	33597	16.29
CCG	1558	0.350	25122	16.12
AAT	1477	0.332	26508	17.95
Tetranucleotide	1026	0.230	23372	22.78
AAAG	249	0.056	5784	23.23
AAAC	164	0.037	3484	21.24
AAGG	84	0.019	1912	22.76
AACG	82	0.018	1844	22.49
Others	447	0.100	10348	23.15
Pentanucleotide	220	0.049	5980	27.18
AAACC	40	0.009	1195	29.88
AAAAG	36	0.008	935	25.97
AGAGG	21	0.005	570	27.14
AAAAC	20	0.004	515	25.75
Others	103	0.023	2765	26.84
Hexanucleotide	419	0.094	13704	32.71
AAAAAC	42	0.009	1326	31.57
ACCTGC	29	0.007	870	30.00
AAGGTG	23	0.005	690	30.00
AGGAGT	19	0.004	570	30.00
Others	306	0.069	10248	33.49
Total	445139	100	9411582	21.14

### Analysis of polymorphic markers

To construct the map, a total of 8,497 markers were screened with the DNA from the 01–88 and 02–12 parental lines. Fifteen percent (1,274) of the markers were polymorphic (Table 4), including 417 (12.34%) SSRs and 646 (29.36%) SNPs. The SNPs were more useful than the SSRs for map construction.

**Table 4:** Characteristics of the primers used in this study

**Sources of primers**	**Number of primer pairs**	**Number of polymorphic primer pairs**	**Frequency of polymorphism primer pairs**
Sequencing of *B. oleracea*	3378	417	12.34
Resequencing of *B. oleracea*	2200	646	29.36
Public markers of *B. oleracea*	551	52	9.44
Associate Researcher Zhuang in IVF CAAS	1080	85	7.87
Professer Liu Kede in Huazhong Agricultural University	268	15	5.60
BAC database of *B. rapa*	292	19	6.51
Sequencing of *B. rapa*	728	40	5.49
Total	8497	1274	14.99

### Skewed segregation of markers

Because of differences among genotypes in their responsiveness to microspore or anther culture, most of the *Brassica* linkage maps based on DH populations contain markers with skewed segregations [34-36]. In the present study, 1,227 of the 1,274 markers (96.31%) that were polymorphic between the two parental lines were assigned to linkage groups. Of those, 449 (33.69%) had skewed segregation patterns, including 203 (33.72%) SSRs and 246 (39.36%) SNPs (Table 5). There were segregation distortion regions (SDRs) in all of the linkage groups except C03 and C04, the markers in the SDRs were randomly distributed. C05, C06 and C09 with five SDRs each had the highest numbers. The longest SDR, with 76 markers, was on C02, where it covered 30.07% of C02. The shortest SDR with only 10 markers was in C05 and C06 (Table 6). 

**Table 5:** Characteristics of the molecular markers used in mapping

**Molecular markers**	**Number of polymorphism primers**	**Number of linked markers**	**Number of unlinked markers**	**Frequency of unlinked markers (%)**	**Number of distorted markers**	**Rate of distorted markers (%)**
SSR	628	602	26	4.14	203	33.72
SNP	646	625	21	3.25	246	39.36
Total	1274	1227	47	3.69	449	36.59

**Table 6:** Distribution of markers in the segregation distortion regions in the linkage groups

**Linkage groups**	**Number of distorted markers**	**Number of SDRs**^**a**^	**Number of the longest SDRs**	**Distance of the longest SDRs (cM)**
C01	86	4	44	12.37
C02	95	3	76	47.66
C03	16	0	0	0
C04	4	0	0	0
C05	49	5	10	4.08
C06	38	5	10	23.17
C07	26	1	16	11.26
C08	63	3	42	15.45
C09	72	5	37	20.25
Total	449	26	235	134.24

^a^SDRs, segregation distortion regions.

### Construction of the high-density genetic map

**Figure 2:**
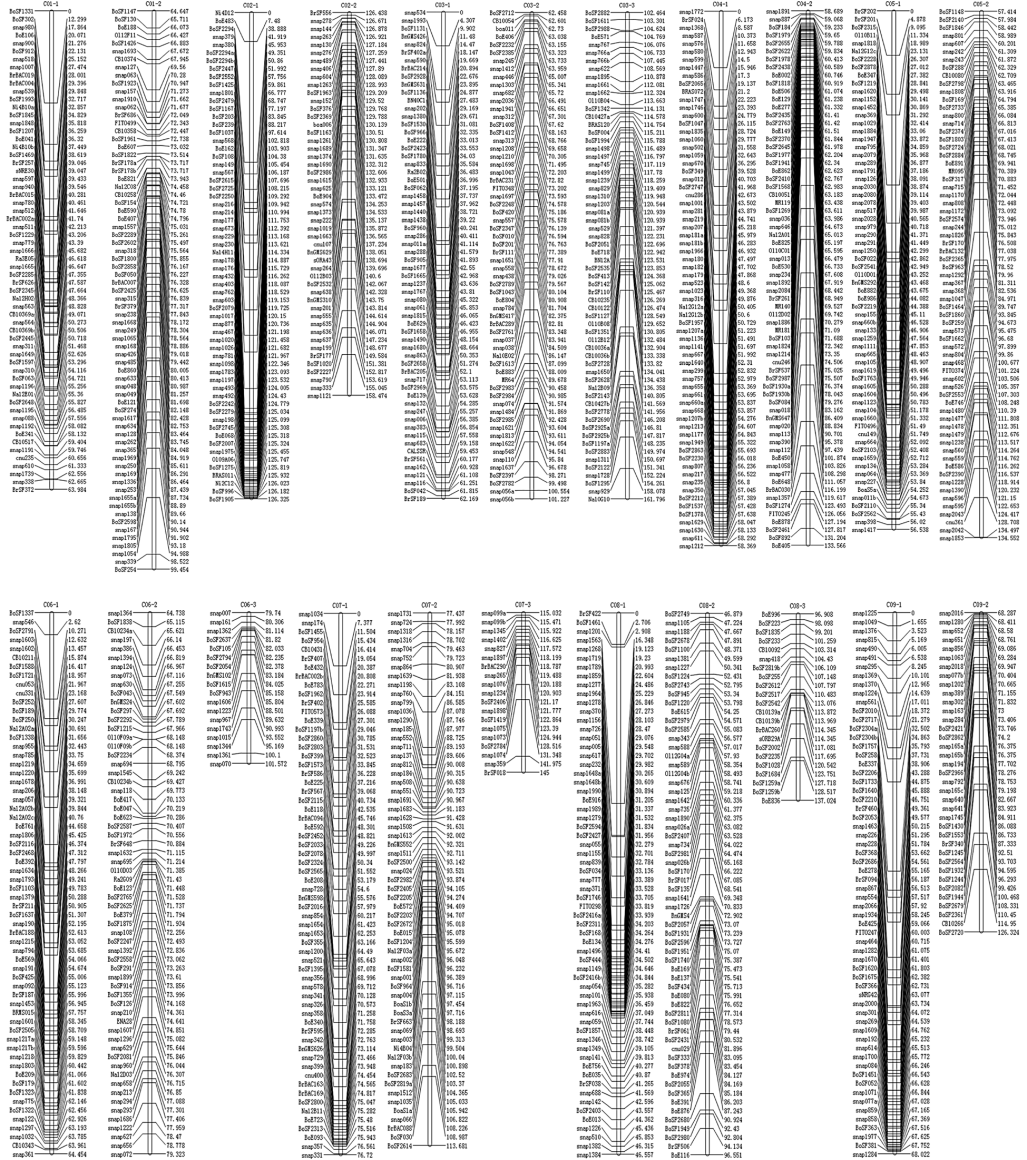
High-density linkage map with 9 linkage groups assigned as C01-C09.

The genetic map that was constructed includes 1,227 markers (602 SSRs and 625 SNPs) assigned to nine linkage groups designated C01–C09 (Figure 2 and Additional file 1), the same as the number of haploid cabbage chromosomes (2n = 2x = 18,n = 9). Only 47 markers (3.69%) were not linked on the map. The map spanned a total of 1197.9 cM. Because the cabbage genome size is approximately 603 Mb [37], the current map represents average genetic and physical intervals of 0.98 cM and 503.3 Kbp per marker, respectively, making it the most saturated linkage map for *B. oleracea* to date. 

**Figure 3:**
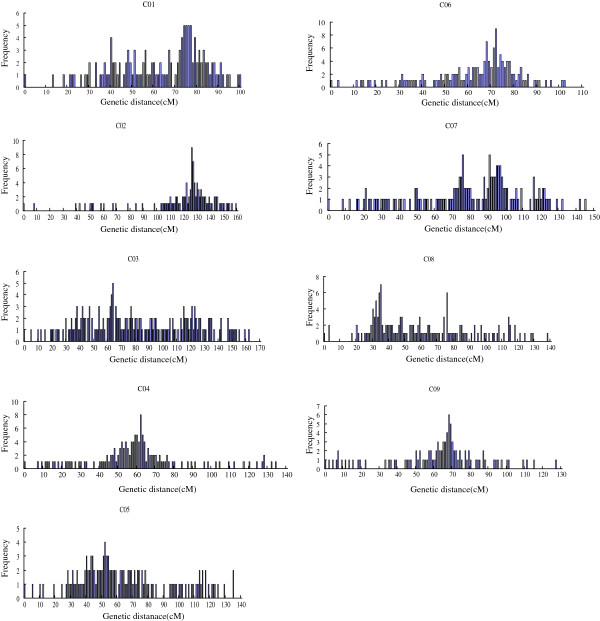
x-axis indicates position in each linkage group in 1cM interval and the y-axis indicates number of markers in the 1cM bin.

The largest linkage group (C03) contained the largest number of markers (186) and spanned 161.8 cM, while the smallest linkage group (C09) contained the smallest number of markers (99) and spanned 126.3 cM. The average distance between adjacent markers went from 0.72 cM (C06) to 1.43 cM (C02). The distribution of SSRs ranged from 43 (C09) to 99 (C03), and the distribution of SNPs ranged from 56 (C09) to 87 (C03). Seven gaps with a distance >5 cM between adjacent markers were spread across C02, and seven gaps with distances >10 cM between adjacent markers occurred on six of the linkage groups: C01, C02, C07, C08 and C09 each with one gap, and C06 with two gaps (Table 7). The results show that the marker loci were unevenly mapped in nine linkage groups (Figure 3).

**Table 7:** **Distribution of molecular markers on the *B. oleracea *high-density genetic map**

**Chromosome**	**Length (cM)**	**Number of markers**	**Number of SSRs**	**Number of SNPs**	**Average distance between two markers (cM)**	**Number of gaps — D**^**a**^**(cM)**	
						**5 < D < 10**	**D > 10**
C01	99.5	132	68	64	0.75	1	1
C02	158.5	111	48	63	1.43	7	1
C03	161.8	186	99	87	0.87	1	0
C04	133.6	136	64	72	0.94	3	0
C05	134.6	133	52	81	1.01	3	0
C06	101.6	142	72	70	0.72	1	2
C07	145.0	142	70	72	1.01	1	1
C08	137.0	146	86	60	0.94	1	1
C09	126.3	99	43	56	1.28	0	1
Total	1197.9	1227	602	625	0.98	18	7

^a^D, gap distance between adjacent markers.

### Distribution of markers along linkage groups

Significant deviations from the random distribution of markers were observed for marker intervals of 1 cM, 2.5 cM, 5 cM, 10 cM, 20 cM, and 40 cM. For a 1 cM interval, the significant deviation (P < 0.001) was shown in Figure 3, indicating that the markers were not randomly distributed in the *Brassica oleracea* linkage groups. Marker distribution for other intervals (2.5 cM, 5 cM, 10 cM, 20 cM, and 40 cM) also showed clustering of markers (P < 0.001) along linkage groups. The independent analysis for testing the random distribution of SSR (P < 0.001) and SNP (P < 0.001) markers indicated deviations from the random distribution.

**Figure 4:**
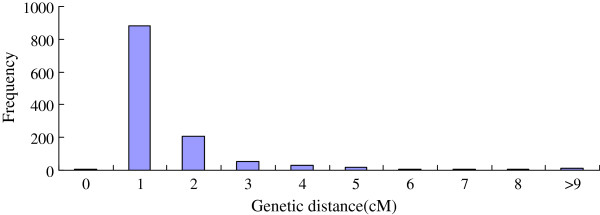
Distribution of map distance between two adjacent mapped makers.

The distance between two adjacent markers on the linkage groups varied from 0 to 31.4 cM, with an average distance of 0.98 cM between two adjacent markers (Figure 4; Table 7). This distance distribution reveals a strong skewness (P < 0.001), further indicating the non-random distribution of the markers along the linkage groups (Figure 4). Among the 1,218 intervals on 9 different linkage groups, 886 intervals were smaller than 1 cM (72.2%), and 24 intervals were larger than 6 cM (2.0%).

## Discussion

### Construction of high-density linkage map of *B. oleracea*

This study was concerned with the construction and comprehensive analysis of a high-density linkage map of *B. oleracea.* The map spans 1197.9 cM and is divided into nine linkage groups corresponding to the number of *B. oleracea* chromosomes, with an average distance of 0.98 cM between adjacent markers. Significantly, of the 1,227 mapped markers, 1,063 (86.63%) were sequence-based markers for *B. oleracea*.

The main purpose of constructing the map was to anchor and orient scaffolds onto the pseudochromosomes for the *B. oleracea* sequencing project. Approximately 83% of the *B. oleracea* genome has been assembled using this high-density linkage map, and the results will be published in a future paper.

Ideally the average distance between adjacent markers should be short and the markers should be evenly distributed throughout the genome. However, many of the markers on the linkage maps of tomato [38], barley [39] and maize [40] have been reported to be clustered. Similarly, the markers on the *B. oleracea* genetic linkage map created in the present study are not evenly distributed.

SSRs are efficient anchor markers with high levels of polymorphism and single locations. They can be used to integrate different linkage maps and chromosomes. SSRs also make good probes for fluorescent situ hybridization (FISH) to integrate genetic and cytogenetic maps.

The main reason why the distribution of molecular markers is uneven is that some chromosomal regions in the parental lines lack polymorphisms. Therefore, the development of mapping populations from different crosses and the use of new molecular markers, including SNPs and SSRs are effective ways to fill in gaps between makers to obtain saturated genetic maps.

### Comparison with published maps of *B. oleracea*

A total of 18 different genetic linkage maps of *B. oleracea* have been constructed (Table 8). The first substantial linkage map based on an F_2_ cross between cabbage and broccoli, contained a total of 258 RFLP markers and covered 820 cM [9]. Since then, the construction of molecular genetic linkage maps of *Brassica* has developed rapidly. Kianian and Quiros [41] developed a map from an F_2_ that consisted of 92 RFLP and 16 isozyme markers covering over 747 cM in 11 linkage groups. A linkage map consisting of 159 RFLP markers with a total distance of 921 cM was constructed from an F_2_ cross between cabbage and broccoli [8]. A *B oleracea* genetic map consisting of 279 molecular markers and one phenotypic marker distributed along nine linkage groups (C01–C09) with a total distance of 891.4 cM and a marker density of 3.2 cM/marker has also been published [42]. The highest density *Brassica oleracea* linkage map developed by Gao et al. [43] comprised of 1,257 markers. Although this linkage map contained more markers than ours, most markers on their map were SRAP markers, which could not be transferred to other linkage map easily. The map constructed in this study comprised of markers which were SSR and SNP markers that were easy to transfer to other linkage maps. 

**Table 8:** **Comparison of the newly constructed map with previously published genetic linkage maps of *B. oleracea***

	**Population**	**Cross combination**	**Types of markers**	**Number of markers**	**Map length (cM)**	**References**
1	F_2_	Broccoli × Cabbage	RFLP	258	820	[9]
2	F_2_	Cabbage × Chinese cabbage	RFLP	201	1112	[44]
3	3 F_2_	Collard × Cauliflower Collard × Broccoli Kale × Cauliflower	RFLP isozyme	108 (integrated)	747	[41]
4	F_2_	Cabbage × Broccoli	RFLP	112	1002	[45]
5	DH	Broccoli × Chinese kale	PFLP	303	875	[7]
6	BC_1_	Chinese kale × Broccoli	RFLP RAPD isozyme	138	747	[46]
7	F_2_	Cabbage × Broccoli	RFLP RAPD	159	921	[47]
8	DH	Cabbage × Broccoli	RFLP AFLP	92	165	[48]
9	F_2_	Cabbage × Chinese cabbage	RFLP RAPD STS SCAR Phenotypic isozyme	310	1606	[49]
10	F_2_	Collard × Cauliflower	RFLP	167	1738	[50]
11	F_2_	Cabbage × Kale	RAPD RFLP isozyme	124	823.6	[51]
12	2DH	Chinese Kale × Broccoli Cauliflower × Brussels sprouts	RFLP AFLP	547 (integrated)	893	[52]
13	F_2_	Chinese kale × Cabbage	RAPD	96	555.7	[53]
14	F_2_	Cabbage × Broccoli	AFLP RAPD SSR	405	731.9	[54]
15	F_2_	Kale × Broccoli	RFLP	199	1226.3	[55]
16	F_2_	Cauliflower × Cauliflower	AFLP NBS	255	668.4	[56]
17	F_2_	Broccoli × Cauliflowe	SRAP SSR	1257	703	[43]
18	DH	Chinese cabbage × Broccoli	RFLP SSR	279	891.4	[42]
	DH	Cabbage × Cabbage	SSR SNP	1227	1197.9	This study

The map produced in the present study contains the second largest number of transferable markers out of the 18 genetic linkage maps constructed thus far. The average genetic and physical intervals, however, are the shortest at 0.98 cM and 503.3Kb per marker, and the number of transferable markers on the map is more than on all the previously published genetic maps. In addition, because the mapping population was a doubled haploid (immortal) produced from a cross between cabbage varieties the map will enhance the efficiency of cabbage breeding, compared to the other maps that were produced from crosses between different varieties such as cabbage and broccoli, and cauliflower and kale. Therefore, this newly constructed map is not only important for research on the related characteristics of cabbage, but it will also contribute to the exchange of materials between laboratories and successive research in the future.

### The reason for segregation distortion

Segregation distortion is defined as the frequencies of genotypes in offspring that do not conform to those predicted by the classical Mendel's law of inheritance. Genetic mapping studies have demonstrated that this phenomenon occurs in many species, including maize [57,58], rice [59,60] and cherimoya [61]; however, the cause of this marker skewing is still debated.

Skewed segregation of markers affects recombination values between markers which results in decreased accuracy of genetic maps and QTL mapping. The extent of skewness is related to the type of markers, the mapping population that was used, and the genetic relationships of the parents. In general, the skewness of co-dominant markers is less than dominant ones [62]. The skewed segregation ratio of recombinant inbred lines is higher than backcross populations (BC) and doubled haploid populations (DH). The F_2_ population has the lowest marker skewness. A low frequency of skewness implies that the parental genetic relationship is close [41].

Lyttle [63] suggested that skewed segregation was one of the engines of evolutionary processes, and that it may be related to the selection of gametophytes or sporophytes. Faure [64] proposed many possible reasons for this phenomenon: (1) the loci on chromosomes are not homologous or translocated, which impacts negatively on synapses in meiosis; (2) different selectivities of gametophyte and sporophyte; (3) interactions between adjacent and linked loci; and (4) non-homologous recombination, gene conversion, and/or transposon from parents [65]. Environmental factors and perhaps other factors may also have to be considered.

Skewed markers may be distributed among linkage groups either as individuals or as clusters. The individually segregated loci occur because of the emergence of systematic segregation [66] and are caused by point mutations. Often distorted markers are linked in clusters, suggesting that there has been selective process of gametophytes or sporophytes [61].

In the current study, we identified 26 distorted regions on linkage groups C01, C02, C05, C06, C07, C08 and C09. The SDRs were distributed as clusters, which is similar to the results of studies on other crops. The highest numbers of distorted SDR markers were found near the middle of the linkage groups, and the numbers gradually reduced towards the ends. In summary, studies of the linkage maps of rice, maize and other crops have shown that SDR loci may be linked to sterility genes and pollen suppressed genes which, in turn, affects the selection of partial gametophytes or sporophytes. It is important to note that while the phenomenon of skewed segregation was observed in *B. oleracea*, it requires further investigation.

## Conclusions

The high-density linkage map of *B. oleracea* L. var. *capitata* was constructed with the aim of using it to anchor the assembled scaffolds to pseudochromosomes, and the assembly of the cabbage genome sequence (to be published soon) has been completed using this map. The map will also provide a useful resource for positional cloning, molecular breeding, and integration of information of genes and traits in *B. oleracea*.

## Competing interests

The authors declare that they have no competing interests.

## Authors' contributions

WW isolated samples, generated the SSR and SNP markers, analyzed marker data, and wrote and revised the manuscript. SH analyzed the sequencing and re-sequencing data, designed the SSR and SNP markers, and revised the manuscript. YL designed the study and critically reviewed the manuscript. ZF designed the study. LY, MZ and YZ generated and managed the plants. WH and SL coordinated and designed the study. SY developed the DH population. JS and AZ performed some of the experiments. All the authors have read and approved the final manuscript.

## Supplementary Material

Additional file 1 Details of the positions, genetic distances, names, types and sequences of all the markers for the linkage groups on the cabbage linkage map. Click here for file
